# The Portland Meeting of the A. M. A.

**Published:** 1905-06

**Authors:** 


### The Portland Meeting of the A. M. A.

THE time appointed for the American Medical Association meeting at Portland, Ore., July 11 to 14, 1905, is near at hand. Those who have not already done so must soon make choice of routes of travel to and from the meeting. The rates are more favorable than ever and it is expected from the reports from all parts of the country that the attendance will be unusually large.

The grand scenery of the Rocky mountain district, the speed and luxury of railway trains, the Lewis and Clark Exposition at Portland, and finally the scientific and social features of the meeting itself, all conspire to make it an attractive summer holiday for physicians and their families. Never before have the rates been so low, nor have the facilities been so comfortable for a transcontinental journey as now. It is the duty of every American of culture to become familiar with his own country. The trip now in prospect is both instructive and healthful.

In our advertising pages will be found information as to routes. The Wabash, the Northwestern, the Union Pacific, and the Wisconsin Central are all in the field with attractive schedules. We advise our readers to look up the subject at an early day, lest some of the choice selections be made by those who appreciate the advantages of so doing before they are appropriated.

---

THE associated alumni of Buffalo, organised in the interest of university extension, at a recent meeting held at the university club, created the following committees: executive committee, John Lord O'Brian, chairman.

To investigate cost of maintenance of arts and technical courses in the smaller colleges: J. N. Larned, chairman; Francis Almy, Frederick J. Shepard, G. A. Ricker, Harry L. Taylor.

To report on demand for higher education in Buffalo among men who cannot attend out-of-town colleges: Arthur Detmers, chairman; Frank S. Fosdick, Henry P. Emerson, M. A. G. Meads, Frederick A. Vogt, Daniel Upton.

Publicity: Charles P. Norton, chairman; Edwin S. Fleming, Edward D. Strickland, Frank Hyatt Smith, Frank H. Severance.

Speakers: John Lord O'Brian, chairman; Charles P. Norton, George F. Buck.

President O'Brian of the executive committee was empowered to add to the committee a representative of each college whose Buffalo alumni are not now represented on the committee.

---

Mrs. Louisa Busch, of Buffalo, was lately tried before Justice Childs and a jury in the criminal term of the supreme court, on an indictment charging her with illegally practising medicine. She was defended by Eugene L. Falk. The case had been heard previously in police court and the defendant was released on suspended sentence. The Medical Society of the County of Erie, through its attorney, Mr. John Lord O'Brian, brought the case to the notice of the district attorney, who secured an indictment and conviction. Some of the testimony was amusing.

Part of Mrs. Busch's defense was the swearing of character witnesses. One of them was a Genesee street saloonkeeper, named Fribolin.

"Do you know the reputation of Mrs. Busch for truthfulness, honesty and uprightness?" asked Mr. Falk. The witness hesitated. "Do you know what the neighbors say about her?" asked Mr. Falk. "Sure," readily responded the witness this time, "but I don't believe all I hear."

The jury was out but a short time. A fine of $250 was imposed by Justice Childs. She paid the fine.

Eugene L. Falk, the woman's attorney, criticised the law under which John W. Ryan of the district attorney's staff effected the conviction.

Justice Childs replied that it was a salutary law and that the efforts of the Erie county physicians to have it respected would always receive the coöperation of the courts.

---

Acknowledgment.—The excellent discussion of Dr. Bentz's paper on traumatic tetanus, published in the Journal for May, 1905, was reported by Dr. George F. Cott, of Buffalo, who has so many times shown his staunch friendship for the magazine in similar fashion. We take pleasure in extending to Dr. Cott our best thanks.

